# One-year Observation of Safety of Implantable Collamer Lens V4c Implantation Without Using an Ophthalmic Viscosurgical Device

**DOI:** 10.3389/fmed.2022.790137

**Published:** 2022-02-14

**Authors:** Zhuoyi Chen, Lingling Niu, Jing Zhao, Peijun Yao, Xiaoying Wang, Xingtao Zhou

**Affiliations:** ^1^Eye Institute and Department of Ophthalmology, Eye & ENT Hospital, Fudan University, Shanghai, China; ^2^NHC Key Laboratory of Myopia (Fudan University), Shanghai, China; ^3^Key Laboratory of Myopia, Chinese Academy of Medical Sciences, Shanghai, China; ^4^Shanghai Research Center of Ophthalmology and Optometry, Shanghai, China

**Keywords:** refractive surgery, ICL (implantable collamer lens), myopia, ophthalmic viscosurgical device, new surgical and technical approach

## Abstract

**Purpose:**

To investigate the feasibility and safety of the implantable collamer lens V4c (ICL V4c) implantation without using an ophthalmic viscosurgical device (OVD-free technique).

**Methods:**

This prospective consecutive case study enrolled 118 eyes of 60 patients (15 eyes were of male patients, 103 eyes were of female patients, age 26.19 ± 5.03 years, spherical equivalent −10.05 ± 2.73 D). Eyes were considered for OVD-free or OVD-using ICL V4c implantation based on the maintenance of the anterior segment during the surgery. The follow-up lasted for 12 months. The main measurements were visual acuity and changes in endothelial cell density (ECD) at 12 months.

**Results:**

A total of 75 eyes were included in the OVD-free group and 43 in the OVD group. No infection or other complications were observed in any eye. In the OVD-free group, the safety and efficacy indices were 1.19 ± 0.15 and 1.05 ± 0.20, respectively. 74.5% of the eyes gained one or two lines of corrected distance visual acuity (CDVA), and 25.5% were stable. In the OVD group, the safety and efficacy indices were 1.17 ± 0.17 and 1.03 ± 0.15, respectively; 65.7% of the eyes gained one or two lines of CDVA, and 34.3% were stable. The mean change of ECD was 65.34 cell/ mm^2^ compared to the baseline in the OVD-free group and 25.94 cell/ mm^2^ compared to baseline in the OVD group (*P* = 0.038).

**Conclusions:**

The ICL V4c implantation with an OVD-free technique is a safe and feasible method in eyes with good maintenance of the anterior segment.

## Introduction

Implantation of a Phakic Vision implantable collamer lens with a central hole (ICL V4c) has been proven to be safe and effective for myopia correction, especially for high myopia (1–3). Like other intraocular surgeries, ophthalmic viscosurgical devices (OVDs) are often used as protective devices for the corneal endothelium in ICL implantation. During traditional surgery, the OVD is injected before ICL implantation, and then washed off after the ICL is placed (4–6). However, OVD shortcomings may also appear in clinical practice, including prolonged surgery duration, increased number of surgical steps, and increased rate of OVD-related complications such as anterior capsule cataract and pupillary block glaucoma (7–10). After decades of development, some surgeons have begun trying to decrease the usage of OVD in ICL implantation, and the established works have proven the safety of this method (11, 12). Hence, it is worthwhile to investigate the possibility of ICL implantation without any OVDs.

Since the ICL V4c was introduced into China in 2014, the authors of this article gradually applied the implantation with the one-step technique (the OVD was injected once, only after the implantation) (11), and then attempted to perform the surgery without OVD. This procedure, referred to as the “OVD-free technique”, implanted the ICL into the posterior chamber through the cornea incision directly, and only selectively injected the balanced salt solution (BSS) afterwards. In this study, we aimed to investigate the feasibility and safety of this technique. This is the first prospective study to do so.

## Patients And Methods

### Ethics and Informed Consent

This study was approved by the Ethical Committee of the Eye & ENT Hospital, Fudan University. All participants provided written informed consent.

### Patients

In this prospective consecutive case study, patients between 18 and 45 years of age who intended to undergo ICL V4c implantation for myopia correction were enrolled. Patients with anterior chamber depth (ACD) <2.8 mm, endothelial cell density (ECD) <2,000/mm^2^, or other ocular diseases (cataract, glaucoma, and retinal detachment) and systemic diseases were excluded. The OVD-free technique was selectively applied depending on the maintenance of the anterior chamber during surgery. All eyes were divided into the OVD-free or OVD group based on the use of the OVD-free technique.

### Examinations

Pre- and post-operative examinations included uncorrected distance visual acuity (UDVA), corrected distance visual acuity (CDVA), corneal endothelium, and intraocular pressure. The corneal endothelium was evaluated using a non-contact specular microscope (Tomey, Japan). The examinations were automatically measured by the instrument. Measurements included ECD, coefficient of variation (CV), and percentage of hexagonal cells (6A%), which were directly interpreted from the results. Intraocular pressure was measured using a non-contact tonometer (Canon, Japan). The preoperative anterior segment depth, corneal thickness, and postoperative vault were measured using the Pentacam (Oculus Optikgerate GmbH, Wetzlar, Germany).

### Surgical Procedure

**Figure 1:**
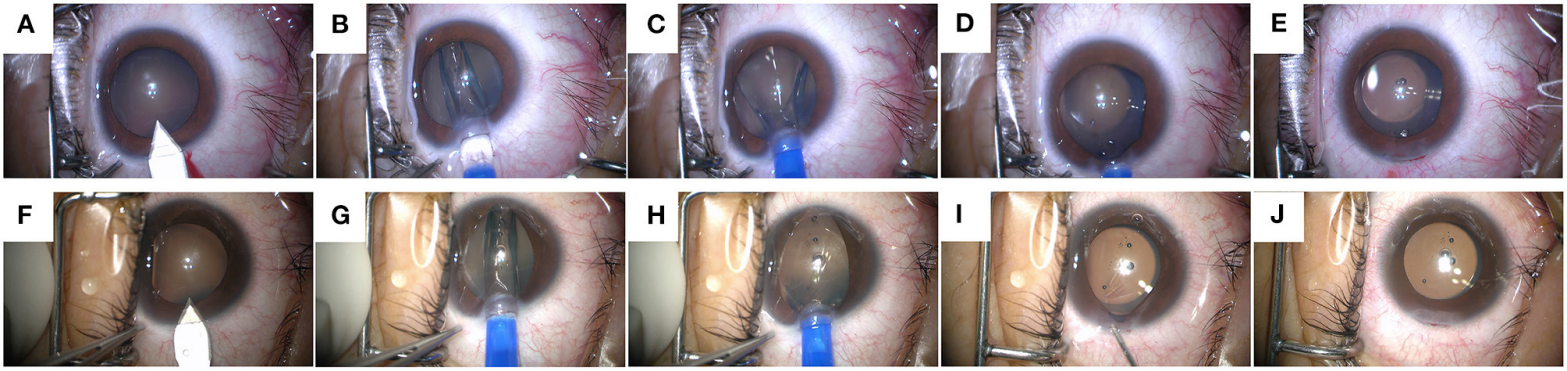
The surgical steps of Implantable Collamer Lens V4c implantation with an ophthalmic viscosurgical device-free (OVD-free) technique. Case 1 **(A–E)**: **(A)** Incision creation. **(B–D)** ICL-V4c injection and placement. **(E)** The status at the end of the surgery. Case 2 **(F–J)**: **(F)** Incision creation. **(G,H)** ICL-V4c injection and placement. **(I)** Balanced salt solution injection. **(J)** The status at the end of the surgery.

All surgeries were performed by the same experienced surgeon (XTZ). 0.5% levofloxacin (Cravit; Santen, Osaka, Japan) was administered 3 days before the surgery. Tropicamide phenylephrine (Santen, Osaka, Japan) was applied 15 min preoperatively. Oxybuprocaine hydrochloride 0.4% (Santen, Osaka, Japan) was used two times for topical anesthesia. A 3-mm temporal incision was made at the beginning and ICL V4c was subsequently injected into the posterior chamber using an injector cartridge. For eyes in which the incision was perfect, the anterior chamber was normal, and the ICL haptics were placed directly into the ciliary sulcus, the OVD-free technique was applied. In detail, the distal haptics should reach the ciliary sulcus first, the ICL should subsequently be gently pushed to unfold the distal haptics, while constantly injecting the ICL and pushing the proximal haptics into the ciliary sulcus as well. Occasionally the surgeon injected the BSS to help stabilize the anterior chamber (OVD-free group) (Figure 1). For eyes that did not meet the above criteria, a minimum OVD (1% sodium hyaluronate) was injected to facilitate ICL placement. Then, the OVD was washed off (OVD group).

Postoperative medication included 0.5% levofloxacin four times per day for 7 days, 1.0% prednisolone acetate (Pred Forte; Allergan, Irvine, CA, USA) four times per day for 3 days, 0.1% fluorometholone (Flumetholon; Santen, Osaka, Japan) four times per day for 7 days, pranopulin (Senju, Osaka, Japan), and 0.1% sodium hyaluronate (Santen, Osaka, Japan) four times per day for 2 weeks.

### Follow-Up

All examinations were carried out at 1 week, and 1, 3, and 12 months after surgery. IOP was also measured 1 day postoperatively.

### Statistical Analysis

All analyses were performed using the open-source R statistical software (v4.0.1, http://www.R-project.org). Data are expressed as mean ± SD. Frequency counts and percentages of participants within each category were calculated for categorical data. Differences in baseline characteristics between groups were assessed using the unpaired Student's *t*-test or Kruskal–Wallis test for quantitative variables, and the χ^2^ test or Fisher's exact test for qualitative variables. The between-group differences were analyzed using the generalized estimating equation (GEE) method for repeated measurements of two eyes and adjusted for sex, age, and spherical equivalent (SE) of the baseline in the models. All significance tests were two-sided, and statistical significance was set at *P* < 0.05.

## Results

A total of 118 eyes of 60 patients were included, of which 15 were of male patients and 103 were of female. The average age was 26.19 ± 5.03 years. The pre-operative SE was −10.05 ± 2.73 D. Seventy-five eyes were included in the OVD-free group and 43 eyes in the OVD group. Bleeding, infection, cataract, and other complications were not observed during follow-up. Seventy-three percent of the eyes (55 eyes) in the OVD-free group and 85% (35 eyes) in the OVD group completed the last follow-up (Table 1).

**Table 1:** Baseline characteristics of the participants.

**Characteristics**	**OVD-free group (*N* = 75)**	**OVD group (*N* = 43)**
Age (years)	26.09 ± 4.89	26.37 ± 5.31
Corrected distance visual acuity (Logmar)	−0.00 ± 0.04	−0.00 ± 0.05
Spherical equivalent (D)	−10.27 ± 2.60	−9.68 ± 2.94
Anterior chamber depth (μm)	3.21 ± 0.23	3.18 ± 0.25
Corneal thickness (μm)	526.23 ± 29.35	528.30 ± 37.48
Intraocular pressure (mmHg)	15.19 ± 2.52	16.15 ± 2.99
Endothelial cell
Endothelial cell density (cell/mm^2^)	2620.03 ± 239.50	2572.40 ± 211.57
Coefficient of variation (%)	41.50 ± 4.75	41.59 ± 5.09
Percentage of hexagonal cells (%)	39.78 ± 8.32	39.34 ± 7.23

*OVD, ophthalmic viscosurgical device*.

### Safety

**Figure 2:**
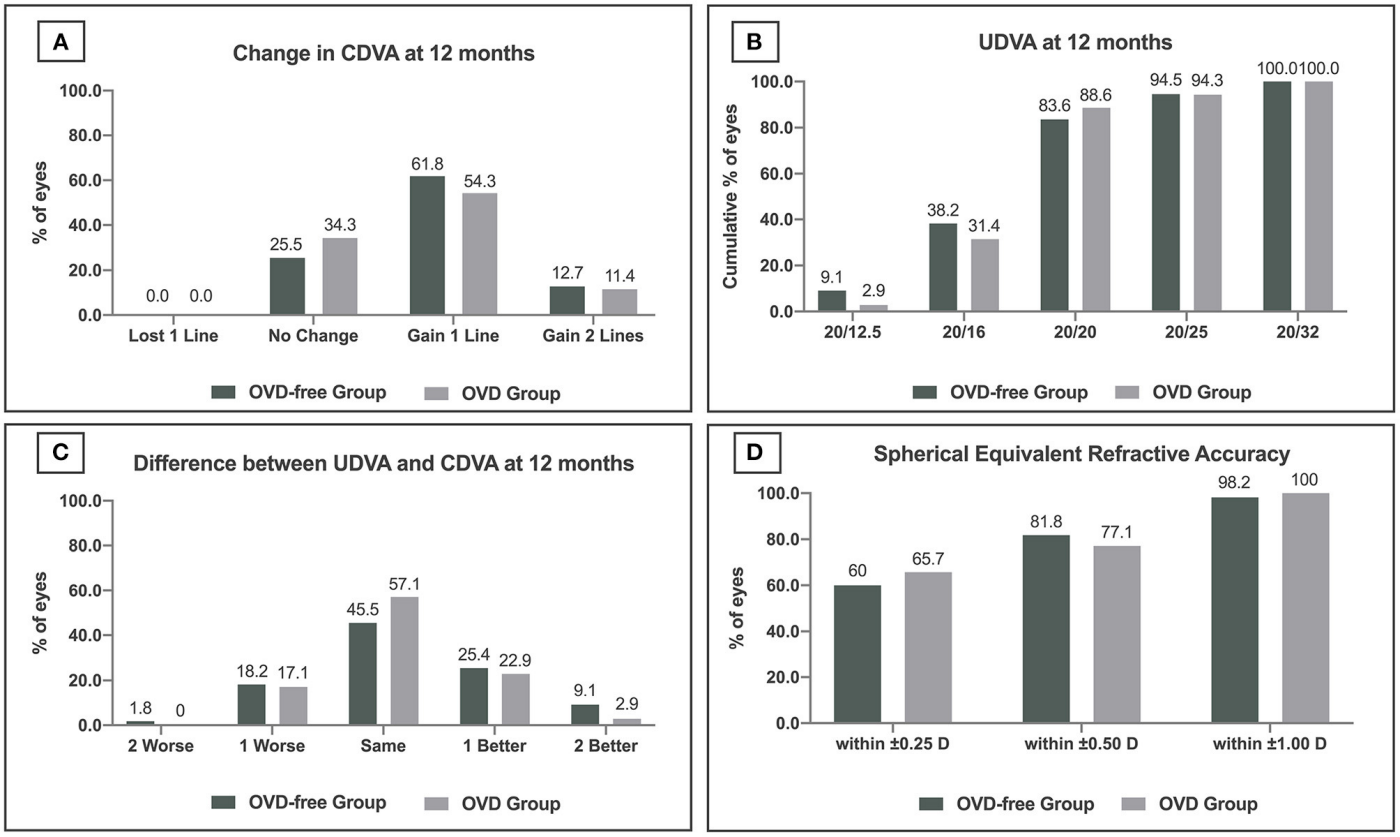
Refractive outcomes at 12 months after the Implantable Collamer Lens V4c implantation in ophthalmic viscosurgical device-free (OVD-free)/OVD group. **(A)** Change in corrected distance visual acuity (CDVA). **(B)** Uncorrected distance visual acuity (UDVA). **(C)** Difference between postoperative UDVA and preoperative CDVA. **(D)** Spherical equivalent refractive accuracy.

At the last follow-up, the safety index was 1.19 ± 0.15 and 1.17 ± 0.17 in the OVD-free group and OVD group, respectively (*P* = 0.357). No CDVA loss was observed in either group. 74.5% of eyes (41 eyes) in the OVD-free group gained one or two lines, and 25.5% of eyes (14 eyes) were stable. 65.7% of eyes (23 eyes) in the OVD group gained one or two lines, and 34.3% of eyes (12 eyes) were stable (Figure 2A). The percentage of eyes gaining CDVA was not significantly different between the two groups (*P* = 0.368).

### Efficacy

The efficacy indices of the OVD-free and OVD groups were 1.05 ± 0.20 and 1.03 ± 0.15 at the last follow-up (*P* = 0.636). 83.6% (46 eyes) and 88.6% (31 eyes) of eyes achieved UDVA ≥20/20 in OVD-free and OVD groups, respectively (*P* = 0.516) (Figure 2B). 34.5% of eyes (19 eyes) achieved UDVA one or two lines better than preoperative CDVA in the OVD-free group. The percentage in the OVD group was 25.8% (9 eyes) (*P* = 0.378) (Figure 2C).

### Accuracy

Sixty-percent (33 eyes), 81.8% (45 eyes), and 98.2% (54 eyes) of eyes in OVD-free group were within ±0.25 D, ±0.50 D, and ±1.00 D of target refraction. The percentages in the OVD group were 65.7% (23 eyes), 77.1% (27 eyes), and 100% (35 eyes), respectively (Figure 2D).

### Corneal Endothelium

**Figure 3:**
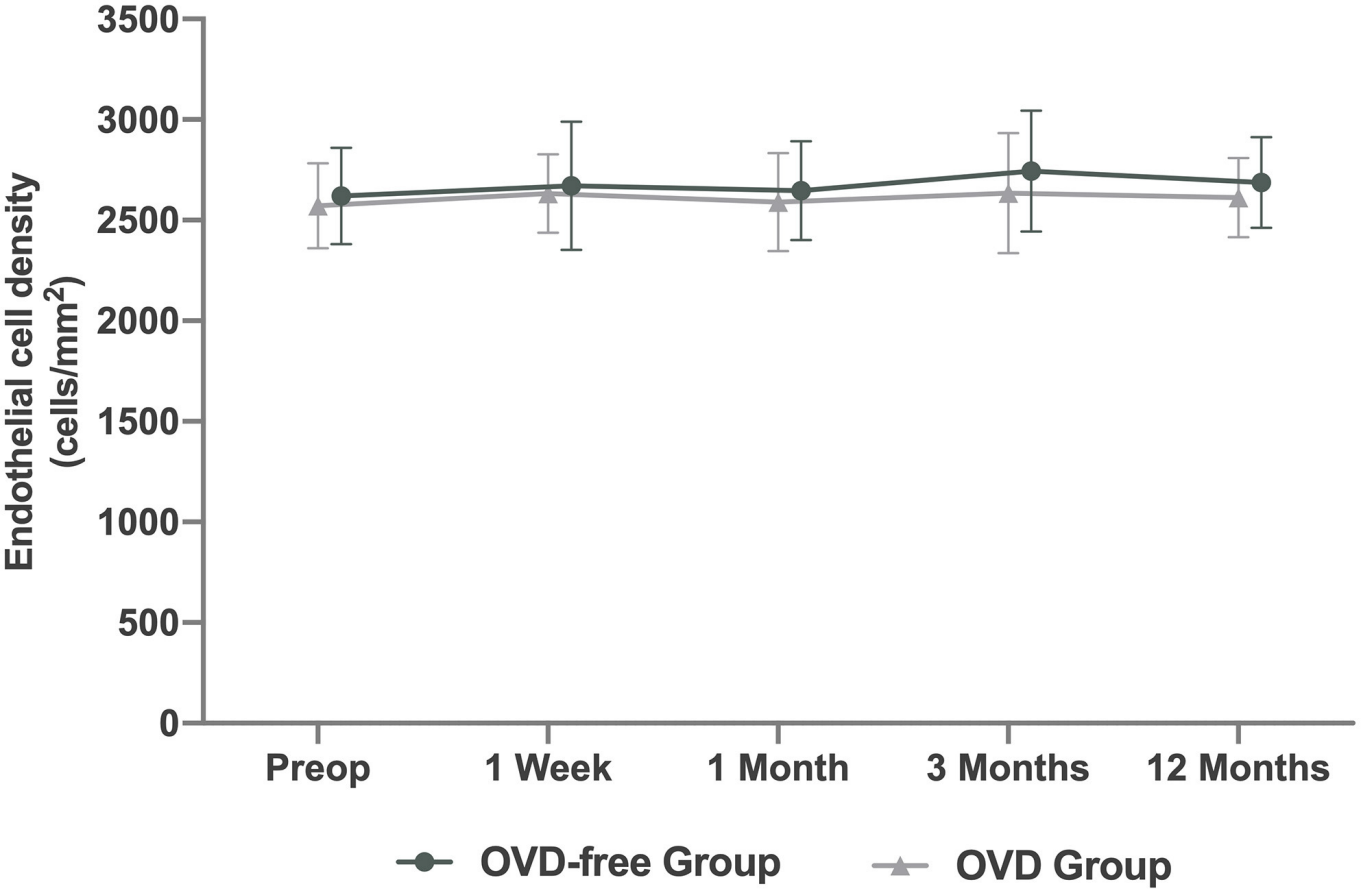
Time course of changes in endothelial cell density (ECD) after Implantable Collamer Lens V4c implantation in ophthalmic viscosurgical device-free (OVD-free)/OVD group.

The preoperative ECD was 2,620 ± 239.50 cell/mm^2^ in the OVD-free group. The ECD change was 65.34 ± 213.47 cell/mm^2^ 12 months postoperatively. The preoperative ECD was 2,572.40 ± 211.57 cell/mm^2^ in OVD group, and the 12-month change was 25.94 ± 163.66 cell/mm^2^. The change in ECD in OVD-free group was 39.40 cell/mm^2^ more than that in OVD group (*P* = 0.038) at 12 months. No significant differences were observed at 1 week, 1 month, and 3 months. The comparison of changes in CV and 6A% showed that the two groups were not significantly different during the whole follow-up, except for the 6A% at 3 months (OVD-free group: 3.38 vs. OVD group: 5.69, *P* = 0.038) (Table 2, Figure 3).

**Table 2:** Changes in endothelial cells and intraocular pressure in OVD-free/OVD group.

**Variable**	**OVD-free group**	**OVD group**	***P*-Value**
ECD (cell/mm^2^)
1 week	50.54 ± 208.70	51.39 ± 149.82	0.8870
1 month	37.95 ± 173.24	44.50 ± 144.84	0.9355
3 months	87.26 ± 260.86	26.70 ± 280.39	0.1389
12 months	65.34 ± 213.47	25.94 ± 163.66	0.0382^*^
CV (%)
1 week	−3.50 ± 4.07	−3.11 ± 3.71	0.8024
1 month	−1.17 ± 4.84	−1.85 ± 3.25	0.3188
3 months	−1.22 ± 6.84	−2.75 ± 6.21	0.1782
12 months	−2.16 ± 4.64	−1.33 ± 3.69	0.5029
6A (%)
1 week	5.45 ± 5.58	6.44 ± 7.05	0.4996
1 month	2.83 ± 6.96	2.80 ± 5.04	0.5034
3 months	3.38 ± 8.35	5.69 ± 8.53	0.0380^*^
12 months	2.93 ± 6.22	1.48 ± 4.16	0.2503
IOP (mmHg)
1 day	0.73 ± 2.48	−0.05 ± 3.57	0.8676
1 week	0.30 ± 3.96	−0.57 ± 3.28	0.6023
1 month	−1.43 ± 2.36	−1.90 ± 3.09	0.9592
3 months	−1.10 ± 2.77	−1.60 ± 2.99	0.2118
12 months	−0.60 ± 1.93	−1.64 ± 2.80	0.0125^*^

* *p < 0.05*.

*OVD, ophthalmic viscosurgical device; ECD, endothelical cell density; CV, coefficient of variation; 6A, percentage of hexagonal cells; IOP, intraocular pressure*.

### IOP and Vault

No abnormal elevation of IOP was observed in either group throughout the follow-up period. The IOP changes in the two groups are shown in Table 2. No excessive or insufficient vault was observed at the 12 month follow-up. The vault of the OVD-free group was 500.83 ± 171.02 μm (range: 200–1,110 μm), and that of the OVD group was 493.57 ± 176.09 μm (range: 200–980 μm) (*P* = 0.844).

## Discussion

ICL implantation is gradually trying to minimize the use of OVDs. As the safety and efficacy of the minimum OVD method has been proven, this study further investigated the safety of ICL V4c implantation with an OVD-free technique on refractive outcomes, corneal endothelium, and IOP.

The 12-month refractive outcomes showed that the OVD-free technique was safe, efficacious, and accurate. In the established 12-month observation of ICL V4c implantation with the conventional use of OVD, Niu et al. (13) demonstrated that 44% of 39 eyes with high myopia gained CDVA after surgery. Similarly, 38 and 4% of 294 high myopic eyes in Kamiya et al.'s. (14) work gained one and two lines of CDVA, respectively, and the UDVA of 97% eyes reached 20/20 or more. In the 12-month studies of ICL V4c implantation with minimum OVD, Chen et al. (12) followed 147 eyes, and the UDVA of 88% eyes was 20/20 or more. And 80 and 98% of eyes were within ±0.50 D and ±1.00 D of target refraction. The results of the OVD-free group were in agreement with those of the above studies. Meanwhile, the OVD-free group showed similar outcomes compared to the OVD group at safety (the percentage of eyes gaining CDVA), efficacy (the percentage of eyes with UDVA 20/20 or more), and accuracy (percentage of eyes within ±0.50 D and ±1.00 D of target refraction), which indicated that the OVD-free technique could also achieve good refractive outcomes.

One important concern in eliminating the use of OVDs is the probable damage to the corneal endothelium. However, the difference in the 12-month ECD change between the OVD-free and the OVD group (65.34 ± 213.47 cell/mm^2^ vs. 25.94 ± 163.66 cell/mm^2^, *P* = 0.038) indicated that the OVD-free technique did not have a negative effect on endothelial cells. Different degrees of increase or decrease in ECD at 12 months after ICL implantation have been described previously. The average change in ECD in Chen et al.'s. (12) study of the minimum OVD method was −42 cell/mm^2^. In Kamiya et al.'s. (14) observation of high myopia with conventional ICL implantation, the ECD increased from 2776.3 to 2796.2 cell/mm^2^. The ECD changes in the above studies were less than those in the OVD-free group in this study. A possible reason is that the OVD-free technique leads to the most simplified intraocular steps, which mostly reduce the involvement of the corneal endothelium. The protective effect of OVD on the endothelium is built on the basis of multiple intraocular steps. Hence, this protection is important for cataract surgery, after which the OVD is adapted for ICL implantation. In fact, ICL V4c implantation did not include steps to remove the cataract. Moreover, the premise of performing the OVD-free technique was that the ICL was placed in the posterior chamber directly, which combined the ICL injection and placement into one step, minimizing the intraocular step and its damage to the corneal endothelium. Therefore, experienced surgeons trying to perform ICL implantation with an OVD-free technique under certain circumstances could achieve a better effect on the corneal endothelium. However, the follow-up lasted for only 12 months. Longer-term observation of this technique would be beneficial in the future.

Furthermore, this study investigated the IOP change after the OVD-free technique and no abnormal elevation was observed during the 12-month follow-up. In Peng et al.'s. (15) work on OVD-free ICL with anterior chamber irrigation, no significant effect of non-OVD and OVD groups was observed at the 2-year follow-up. In addition, Chen et al. (12) demonstrated that the effect of reducing the OVD on IOP was mainly seen 2–3 h after surgery. Theoretically, minimizing OVD use may eliminate OVD-related elevation of IOP.

It is worth pointing out that the OVD-free technique was selectively performed under these principles. First, the application of this technique should be on the eye with adequate anterior chamber depth. Second, a perfect incision and good maintenance of the anterior chamber during surgery were essential for success. Meanwhile, the surgeon's experience is an important factor as well. In clinical practice, OVD use is still important for safe implantation when the anterior chamber is not good enough or if complications arise during ICL injection and placement.

In conclusion, the ICL V4c implantation with an OVD-free technique is safe and feasible in eyes with good maintenance of the anterior segment. Considering its simplification and clinical outcomes, it is worthwhile to try this novel technique for experienced ICL surgeons.

## Data Availability Statement

The original contributions presented in the study are included in the article/supplementary material, further inquiries can be directed to the corresponding author.

## Ethics Statement

The studies involving human participants were reviewed and approved by the Ethical Committee of the Eye & ENT Hospital, Fudan University. The patients/participants provided their written informed consent to participate in this study. Written informed consent was obtained from the individual(s) for the publication of any potentially identifiable images or data included in this article.

## Author Contributions

The study concept and design, drafting of the manuscript and critical revision of the manuscript were done by ZC, LN, and XZ. Data collection was done by ZC, LN, and PY. Analysis and interpretation of data were undertaken by ZC, LN, JZ, and XW. Supervision was done by XZ. All authors contributed to the article and approved the submitted version.

## Funding

This work was supported in part by the National Natural Science Foundation of China (Grant No. 81770955), Joint research project of new frontier technology in municipal hospitals (SHDC 12018103), Project of Shanghai Science and Technology (Grant No. 20410710100), Clinical Research Plan of SHDC (SHDC2020CR1043B), and Project of Shanghai Xuhui District Science and Technology (2020-015).

## Conflict of Interest

The authors declare that the research was conducted in the absence of any commercial or financial relationships that could be construed as a potential conflict of interest.

## Publisher's Note

All claims expressed in this article are solely those of the authors and do not necessarily represent those of their affiliated organizations, or those of the publisher, the editors and the reviewers. Any product that may be evaluated in this article, or claim that may be made by its manufacturer, is not guaranteed or endorsed by the publisher.
